# Iron, Oxidative Stress, and Haptoglobin Gene Polymorphism in Sickle Cell Disease Patients With Inflammation in Cameroon: An Analytical Cross‐Sectional Study

**DOI:** 10.1155/bri/5303373

**Published:** 2025-12-28

**Authors:** Romaric Tuono De Manfouo, Josué Simo Louokdom, Jean Paul Chedjou, Prosper Cabral Biapa Nya, Wilfried Mbatcham, Claude Tayou Tagny, Constant Anatole Pieme

**Affiliations:** ^1^ Department of Hematology, ‘Faculté de Médecine et des Sciences Biomédicales’, ‘Université de Yaoundé I’, Yaoundé, Center Region, Cameroon; ^2^ Department of Medicine and Biomedical Sciences, Faculty of Heath Sciences, ‘Université des Montagnes’, Bangangte, West Region, Cameroon; ^3^ Department of Biotechnology, ‘Centre de Biotechnologie’, Université de Yaoundé 1’, Nkolbisson, Yaoundé, Center Region, Cameroon; ^4^ Department of Biochemistry, Faculty of Science BUEA, University of Buea, Southwest, West Region, Cameroon, ubuea.cm; ^5^ Department of Biochemistry, ‘Faculté des Sciences’ Dschang, Université de Dschang’, Dschang, West Region, Cameroon; ^6^ Department of Biochemistry, ‘Faculté des Sciences’ Yaoundé, Université de Yaoundé I’, Yaoundé, Center Region, Cameroon

**Keywords:** haptoglobin gene polymorphism, oxidative stress, serum iron, sickle cell disease

## Abstract

**Background and Aims:**

The more severe forms of sickle cell disease (SCD) are highly inflammatory genetic disorders that cause significant oxidative stress. To fight against the free radicals produced, the body has an antioxidant system. In addition, haptoglobin has been reported in several studies as possessing oxidant and inflammatory properties depending on the nature of the genotype. This study hypothesizes that the HP2 allele of haptoglobin may exacerbate oxidative stress in sickle cell patients with inflammation.

**Methods:**

An analytical cross‐sectional study was conducted for 6 months. The patients recruited were those with severe forms of sickle cell anemia, regularly followed at the hematology department of the Yaoundé Central Hospital and the Bafoussam Regional Hospital. The Public Health Research Biotechnology Laboratory (LAPHER‐Biotech) in Yaoundé provided a framework for genotyping haptoglobin by allele‐specific PCR. Next, iron, oxidative stress, and inflammatory parameters were assessed by standard methods, and the statistical software *R* Version 4.1.1. allowed for the data analysis.

**Results:**

Samples from 149 participants were analyzed. Patients with Hp phenotypes 2‐2 had a considerable elevation of reduced glutathione (14.3 μmol/L) compared to those of phenotype Hp 2‐1 (11.2 μmol/L) and genotype Hp 1‐1 (11.8 μmol/L) (*p* = 0.075). Malondialdehyde was significantly higher in patients with Hp phenotypes 2‐2 compared to those with Hp phenotype 2‐1 and Hp phenotype 1‐1 (*p* = 0.008). The oxidative stress index (OSI) was higher in patients with the Hp 2‐2 phenotype than those with the Hp 2‐1 and Hp 1‐1 phenotypes (*p* = 0.008) suggesting that they are more affected by oxidative stress.

**Conclusion:**

This study supports the hypothesis that the Hp 2‐2 phenotype of haptoglobin is associated with an imbalance in the oxidative balance in favor of oxidants, suggesting that the latter is a major contributor to the worsening pathophysiology of SCD.

## 1. Introduction

Sickle cell disease (SCD) can result from homozygosity for the *β* ^s^ allele or codominance of *β* ^*s*^ with another mutation, like *β* ^*C*^, *β* ^0^, or *β* ^+^, and the more severe forms are *β* ^*s*^/*β* ^*s*^ and *β* ^*s*^/*β* ^*C*^ [1]. Sickle cell anemia is a genetic condition characterized by abnormal hemoglobin in the blood. The consequences of this genetic abnormality are multiple, including acute and chronic complications characterized by erythroblastopenia, acute anemia, splenic sequestration, and vaso‐occlusive crises [2]. In addition, other sources of inflammation are described, including the high frequency of hemolysis, which continuously releases hemoglobin and heme into the vascular system, alteration of sickle cell red blood cells in hypoxia, recurrent infections, and vaso‐occlusive crises resulting from tissue hypoxia [3].

Intravascular lysis during sickle cell anemia results in the release of HbS into the vascular bed; the hemolysis product will be a significant release of heme and ferric hemoglobin (*F* ^*e*3+^) (metHb) [4]. Iron from heme acts as a molecular mediator of damage and can activate the pattern recognition receptor 4 (TLR4), which triggers oxidative stress, inflammation, and vaso‐occlusive crises [3]. Sickle cell patients are subject to pathological events in favor of increased free radical generation following the significant activation of pro‐oxidant enzymes, hemolysis, which results in the release of free hemoglobin, heme, and iron; all of the above promote the Fenton reaction and are major contributors to altered mitochondrial respiratory chain activity and red blood cell auto‐oxidation. Moreover, the excessive production of free radicals is a major factor in the increase of cellular oxidative stress, including red blood cells, neutrophils, endothelial cells, and platelets. The above is reported as an aggravating factor of the disease because it causes, for example, a multiorgan vasculopathy in sickle cell patients [5, 6]. To counteract the production of free radicals, the body activates a defense system that includes enzymatic and nonenzymatic components [7]. Furthermore, haptoglobin (Hp), a protein coded by the *HP* gene and involved in redox homeostasis and inflammation, can have different actions depending on the *HP* genotype. One of the functions of haptoglobin is its action in the elimination of iron from the vascular hemolytic environment through its binding to free hemoglobin. In its structure, the molecule is composed of two peptide chains, namely, the alpha and beta chains, linked together by disulfide bonds [8]. Both chains are encoded by the same gene located on chromosome 16q22.2 and are synthesized as a pre‐protein that undergoes post‐translational modifications, followed by proteolytic cleavage of the two subunits [9]. This gene exhibits a common polymorphism characterized by two alleles: the *HP* 1 allele, conserved among species, consists of five exons; in comparison, the *HP* 2 allele is human‐specific and contains seven exons likely, the product of a duplication involving exons 3 and 4 of the *HP* 1 allele [9]. These 2 codominant alleles lead to the existence of 3 distinct phenotypes (Hp 1‐1, Hp 2‐1, and Hp 2‐2) [9]. However, hemoglobin‐binding affinity and plasma concentration vary according to the Hp phenotype, with Hp 2‐2 found to have a reduced overall affinity for Hb compared to other phenotypes [9], reduced antioxidant capacity [3], and clearance rates of *HP*2‐Hb complexes by less efficient macrophages [8]. The consequence of this is an increase in nitric oxide uptake by *HP*2‐Hb complexes [6], with endocytosis by macrophages of *HP*2‐Hb complexes followed by a reduction in downstream anti‐inflammatory signaling [8].

Previous studies have reported associations between the Hp 2‐2 phenotype and certain pathologies. For instance, Cox et al. reported an association between *HP* 2 and worsened malaria infection [10]. Kerchberger et al. also found that the haptoglobin‐2 variant is an aggravating factor in susceptibility to acute respiratory distress syndrome during sepsis [11].

This study hypothesizes that the *HP2* allele of haptoglobin may exacerbate oxidative stress in patients with sickle cell anemia with inflammation. However, limited research has been conducted on oxidative stress and haptoglobin gene polymorphism in Cameroon. Therefore, it is crucial to study the relationship between iron, oxidative stress, and the genetic polymorphism of haptoglobin in SCD patients experiencing inflammation, and explore how these factors would contribute to the worsening of the pathophysiology of the disease.

## 2. Methods

### 2.1. Study Design and Recruitment of the Study Population

The study was an analytical cross‐sectional study and was conducted over 06 months. The patients recruited were those with the more severe forms of SCA in the stable phase, regularly followed at the Hematology Department of the Yaoundé Central Hospital and the Bafoussam Regional Hospital. Participants included both sexes and all age groups, although they were predominantly children. Determination of inflammation allowed participants to be grouped into patients with inflammation and those without.

A structured questionnaire written in the official languages of Cameroon (French and English) including information related to the study was administered to the preselected participants and/or failing that to their legal guardians. The questionnaire was oriented toward information on the identity of the participants, clinical information on the follow‐up of their disease and comorbidities, and the treatments followed. Thus, participants with inflammatory and hemolytic diseases, such as G6PD deficiency, thalassemia, and infections, were excluded from the study. Also, those on iron supplementation, participants on iron chelation treatment, those with vaso‐occlusive crisis, and those who had undergone a blood transfusion less than 3 months ago were not included in this study. In the end, 149 participants were included.

For all participants, child assent and parental consent were obtained. Parents read and signed the informed consent form.

### 2.2. Laboratory Analyses

The Medical Biochemistry Laboratory of the University Hospital of Yaoundé was used as a framework for the Biochemistry analyses from a volume of 04 mL of blood taken in a dry tube without anticoagulant. In addition, the Medical Hematology Laboratory of the same hospital served as a setting for the analysis of 04 mL of blood samples taken from EDTA tubes. At the same time, as blood samples were taken, approximately 40 μL of blood was impregnated onto Whatman paper and then introduced into an envelope containing silica and stored at room temperature (22°C) for further molecular biology analyses. Thus, the Public Health Research Biotechnology Laboratory (LAPHER‐Biotech) in Yaoundé served as a framework for the genotyping of haptoglobin by allele‐specific PCR.

First, the hematological analyses consisted of confirming the status of each of the participants including the performance of quantitative hemoglobin electrophoresis on agarose gel and the determination of the proportions of the hemoglobin fractions according to the “HELLABIO” protocol *(Thessaloniki, Greece).*

Subsequently, the biochemical analyses consisted first of all in the determination of the concentration of the C‐reactive protein according to the method described by the manufacturer of the kit “Genrui” (*Lotus NL B.V. The Hague, Netherlands*), and the assay carried out using a semiautomatic protein analyzer “PA54” calibrated by magnetic card whose operating principle is nephelometry. Next, the serum iron assessment was determined according to the standard methods described by the manufacturer of the “Biolabo” kit (*BIOLABO SAS, Les Hautes Rives, 02160 Maizy, France)* and the assay by spectrophotometric method. Finally, the exploration of oxidative stress was done by the determination of the total antioxidant capacity (TAC) by ferric reducing antioxidant power assay (FRAP) according to the protocol described by Benzie and Strain [12]. The concentrations of malondialdehyde (MDA) and reduced glutathione (Gsh) were evaluated according to the respective protocols of Hundekar et al. [13] and Ellman [14]. The determination of catalase activity was made by deduction from total proteins according to the method described by Sinha [15]. The oxidative stress index (OSI) was deduced by calculation according to the ratio [(MDA/TAC) x 100] [16]. Techniques for exploring stress markers mainly used kinetic and enzymatic methods (*manufacturer Bioquochem, KB03002, Spain)*.

Molecular biology analyses consisted of genotyping of the haptoglobin molecule by PCR using primer pairs and allele‐specific primers, as described by Yano et al. [17]. The steps consisted first of all in the extraction of the DNA by the method with “Chelex 100” [18], followed by amplification as described by Yano et al. [17], and finally, electrophoresis on agarose gel and revelation with ethidium bromide.

Table S1 shows the newly designed primer sequences for haptoglobin genotyping (Given in Supporting Files Table S1) [17].

Table S2 shows the combinations between the primers and the expected size of the amplified DNA fragments in each reaction (*Given* in Supporting Files Table S2).

### 2.3. Data Management and Statistical Analysis

Inflammation was determined from quantitative methods and defined for CRP cutoff values > 10 mg/L as defined by the test kits used and also used by several other authors [19]. *R* Version 4.1.1 statistical software was used to analyze the data previously recorded in the Excel 2013 spreadsheet. Qualitative and quantitative variables were analyzed. Quantitative variables were age, CRP concentration, serum iron, TAC, MDA, reduced glutathione (Gsh), catalase activity, and OSI. Also, biochemical parameters studied have been dichotomized and grouped into “normal, high, or low” according to the scientific literature. Quantitative variables were presented in median and interquartile range (IQR) and qualitative variables in frequency. Median values were compared using the Wilcoxon test, and frequencies were compared using Fisher’s exact test. The significance level was set for *p*‐value < 0.05. A bean plot representation was used to represent the distribution of markers of oxidative stress and the trend line of these points between the different groups (*for the presentation of the results in the supporting information and results*).

### 2.4. Ethical Considerations

This study was carried out in strict compliance with the principles of medical research as set out in the Declaration of World Medical Association [20]. This is how this study was approved by the Centre’s Regional Research Ethics Committee (AUTHORZATION N° E210/CRERSHC2021). The Regional Hospital of Bafoussam (N^°^. 005/L/MINSANTE/SG/DRSPO/HRB/D) and the Central Hospital of Yaoundé (N^°^. 276/21/AR/MINSANTE/SG/DHCY/CM/SM) have been permitted to collect samples, respectively. The Laboratory for Public Health Research Biotechnologies (LAPHER‐Biotech) and the Yaoundé University Hospital have permitted the analysis of the samples.

## 3. Results

### 3.1. Genetic Polymorphism of Haptoglobin in Sickle Cell Patients

The vast majority of the included study population was from West Cameroon and its environs. The study population consisted of 50.3% males (75/149) and 49.7% females (74/149), with a median age of 9 years [4.00–13.0] (Q1–Q3). Table 1 describes the sociodemographics and bioclinical characteristics of the population.

**Table 1:** Description of sociodemographics and bioclinical characteristics of the population.

Variables		Effective	Frequency (%)
Sex:	F	74	(49.7)
	M	75	(50.3)

BMI	Underweight	98	(65.77)
	Normal	51	(34.23)

History of infectious crises:	No	101	(67.8)
	Yes	48	(32.2)

History of anemic crises:	No	71	(47.7)
	Yes	78	(52.3)

History of VOC:	No	68	(45.6)
	Yes	81	(54.4)

History of CVD and stroke	No	146	(98.0)
	Yes	3	(2.01)

VOC/month, Med (IQR)		1.00 [0.00–2.00]	
Age, Med[q1‐q3]		9.00 [4.00–13.0]	
BMI, Mean ± sd		17.2 ± 3.07	

*Note:* Patient of CHY (Central Hospital of Yaoundé) and RHB (Regional Hospital of Bafoussam); M: male; F: female.

Abbreviations: BMI: body mass index; CVD: cardiovascular diseases; VOC: vaso‐occlusive crisis.

### 3.2. Genetic Polymorphism of Haptoglobin in Sickle Cell Patients

The genetic haptoglobin polymorphism described in Table 2 shows a higher frequency of the “*HP*1F” allele (69.8%) followed by “*HP*2” (46.31%). Also, 14.09% of the population had the Hp 2‐2 phenotype.

**Table 2:** Distribution of major sickle cell syndrome patients from RHB and CHY according to the genetic polymorphism of haptoglobin.

Variables (alleles and phenotype)		Hp 1‐1 *N* = 80 *n* (%)	Hp 2‐1 *N* = 48 *n* (%)	Hp 2‐2 *N* = 21 *n* (%)	Total *N* = 149 *n* (%)	*p*‐value
*HP*_2:	No	80 (100)	0 (0.00)	0 (0.00)	80 (53.69)	< 0.001
	Yes	0 (0.00)	48 (100)	21 (100)	69 (46.31)	

*HP*_1S	Non	40 (50.0)	35 (72.9)	21 (100)	96 (64.43)	< 0.001
	Yes	40 (50.0)	13 (27.1)	0 (0.00)	53 (35.57)	

*HP*_1F	No	11 (13.8)	13 (27.1)	21 (100)	45 (30.2)	< 0.001
	Yes	69 (86.2)	35 (72.9)	0 (0.00)	104 (69.8)	

*HP*_2_2	No	80 (100)	48 (100)	0 (0.00)	128 (85.9)	< 0.001
	Yes	0 (0.00)	0 (0.00)	21 (100)	21 (14.09)	

*HP*_1S_1S	No	69 (86.2)	48 (100)	21 (100)	138 (92.62)	0.005
	Yes	11 (13.8)	0 (0.00)	0 (0.00)	11 (7.4)	

*HP* 2_1S:	No	80 (100)	35 (72.9)	21 (100)	136 (91.27)	< 0.001
	Yes	0 (0.00)	13 (27.1)	0 (0.00)	13 (8.7)	

*HP* 1S_1F	No	51 (63.7)	48 (100)	21 (100)	120 (81.08)	< 0.001
	Yes	29 (36.2)	0 (0.00)	0 (0.00)	29 (19.46)	

*HP* 1F_1F	No	40 (50.0)	48 (100)	21 (100)	109 (73.15)	< 0.001
	Yes	40 (50.0)	0 (0.00)	0 (0.00)	40 (26.85)	

*HP* 2_1F:	No	80 (100)	13 (27.1)	21 (100)	114 (76.51)	< 0.001
	Yes	0 (0.00)	35 (72.9)	0 (0.00)	35 (23.49)	

Total		80 (53.69)	48 (32.21)	21 (14.09)	149 (100)	

*Note:* HP = haptoglobin; 1F, 1S, 2 = allele; Fisher test. Significant difference at *p* < 0.05.

### 3.3. Oxidative Profile of the Study Population According to Haptoglobin Phenotypes

Table 3 describes the oxidative profile of the population during the study. The median serum iron in patients is 1.86 mg/L [1.51–2.46] with 87.9% of the population having iron elevation. The median of MDA in patients with the HP 2‐2 genotype, 1.13 μmol/L [0.78–2.13], is significantly higher when compared with those of HP 2‐1 and HP1‐1 patients, 0.46 μmol/L [0.27–0.91] and 0.43 μmol/L [0.28–0.99], respectively (*p* = 0.008). Though not significant, patients with Hp phenotypes 2‐2 have a considerable elevation of reduced glutathione (14.3 μmol/L [11.5–16.8]) compared to those with Hp 2‐1 phenotype (11.2 μmol/L [8.71–14.0]) and Hp 1‐1 phenotype (11.8 [9.83–14.5]) (*p* = 0.075). The “OSI” in patients with Hp phenotypes 2‐2 is 31.4% [19.7–56.9] and 21.9% [15.4–27.2] and 17.7% [12.6–29.8], respectively, in those of phenotypes Hp 2‐1 and Hp 1‐1 (*p* = 0.007) (Figures S1–S5 give a detailed presentation of the different markers according to haptoglobin phenotypes).

**Table 3:** Oxidative profile according to haptoglobin phenotypes.

Variables		*Hp* 1‐1 *N = 80* *n* (%)	*Hp* 2‐1 *N* = 48 *n* (%)	*Hp* 2‐2 *N* = 21 *n* (%)	*p*‐value
Iron, *a* ^*x* *x* *x*^		1.96 [1.46–2.40]	1.82 [1.50–2.74]	1.86 [1.58–1.96]	0.842
Iron	Low	1 (1.25)	0 (0.00)	0 (0.00)	0.109
	High	71 (88.8)	39 (81.2)	21 (100)	
	Normal	8 (10.0)	9 (18.8)	0 (0.00)	

Catalase, *a* ^*x* *x* *x*^		2309 [1757‐3325]	2032 [1589‐3297]	1795 [1607‐3012]	0.316
MDA, *a* ^*x* *x* *x*^		0.43 [0.28–0.99]	0.46 [0.27–0.91]	1.13 [0.78–2.13]	0.008
TAC, *a* ^*x* *x* *x*^		1093 [803‐1303]	849 [778‐1123]	1037 [889‐1180]	0.378
GsH, *a* ^*x* *x* *x*^		11.8 [9.83–14.5]	11.2 [8.71–14.0]	14.3 [11.5–16.8]	0.075
Index OSI, *a* ^*x* *x* *x*^		17.7 [12.6–29.8]	21.9 [15.4–27.2]	31.4 [19.7–56.9]	0.007

*Note:* Iron (mg/L); MDA = malondialdehyde (μmol/L); catalase activity (IU/mg protein); TAC = total antioxidant capacity (μmol TE/g DW); GSH = reduced glutathione (μmol/L); index OSI = MDA/TAC ratio (%); *a* ^*x* *x* *x*^ = Med (IQR); Wilcoxon test. Significant difference at *p* < 0.05 (see Figures S1–S5 for a detailed presentation of the different markers by haptoglobin phenotypes).

### 3.4. Oxidative Profile of the Study Population by Inflammation

The frequency of inflammation was 42.3%. Table 4 describes the oxidative profiles of the study population according to inflammation. The median serum iron in patients is 1.86 mg/L [1.51–2.46] with 87.9% of the population having iron elevation. In addition, there is no significant difference in iron elevation, depending on the presence or the absence of inflammation. Though not significant, the antioxidant capacity is higher in an sickle cell patient 1118 μmol TE/g DW [797‐1300] than in a noninflammatory 959 μmol TE/g DW [784‐1190] (*p* = 0.062). In addition, the other parameters of oxidative stress did not differ significantly according to the inflammatory status of the patients (*A graphical presentation of each of the parameters is given in figures* S6 *–* S9)*.*

**Table 4:** Oxidative profile in the population in the presence or absence of inflammation.

Variables		Presence of inflammation *N* = 63 *n* (%)	Absence of inflammation *N* = 86 *n* (%)	Total *N* = 149 *n* (%)	*p*‐value
Iron, *a* ^*x* *x* *x*^		1.91 [1.52–2.48]	1.84 [1.50–2.31]	1.86 [1.51–2.46]	0.616
Iron	Low	0 (0.00)	1 (1.16)	1 (0.67)	0.774
	High	57 (90.5)	74 (86.0)	131 (87.9)	
	Normal	6 (9.52)	11 (12.8)	17 (11.4)	

Catalase, *a* ^*x* *x* *x*^		2059 [1697‐3113]	2266 [1610‐3322]	2201 [1612‐3214]	0.475
MDA, *a* ^*x* *x* *x*^		0.43 [0.27–1.13]	0.47 [0.31–1.13]	0.45 [0.29–1.13]	0.539
TAC, *a* ^*x* *x* *x*^		1118 [797‐1300]	959 [784‐1190]	1013 [783‐1219]	0.062
GsH, *a* ^*x* *x* *x*^		12.2 [10.3–16.8]	11.8 [8.60–14.0]	12.1 [9.78–14.7]	0.112
Index OSI, *a* ^*x* *x* *x*^		17.6 [12.7–30.8]	22.3 [15.6–31.4]	19.7 [14.7–31.4]	0.116

*Note:* Iron (mg/L); MDA = malondialdehyde (μmol/L); catalase activity (IU/mg protein); TAC = total antioxidant capacity (μmol TE/g DW); GSH = reduced glutathione (μmol/L); index OSI = MDA/TAC ratio (%); *a* ^*x* *x* *x*^ = Med (IQR); Fisher test; Wilcoxon test. Significant difference at *p* < 0.05; see Figures S6–S9 for a detailed presentation of parameters by inflammation.

## 4. Discussion

The purpose of this investigation is to study the relationship among iron, oxidative stress, and the genetic polymorphism of haptoglobin in the sickle cell patient in inflammation, and how oxidative stress and haptoglobin genotype may increase the severity of the disease. Studies on the genetic polymorphism of haptoglobin could provide useful information, as haptoglobin is described as a protein with proinflammatory and anti‐inflammatory, pro‐oxidant, and antioxidant properties, depending on the genotype, and is involved in the mechanism of vascular disease [21]. This study is also the first in Cameroon to present the genetic polymorphism of haptoglobin and to describe how it influences oxidative stress in sickle cell patients in situations of inflammation.

The study population consisted of 50.3% males (75/149) and 49.7% females (74/149). There was therefore no significant link between the type of hemoglobin and sex, and this has a genetic explanation because the transmission of defect is independent of sex. These same observations were made by Doupa et al. and Ousmane et al. [22, 23]. The median age of our participants is 9 [4–13] corresponding to a relatively young age. Houwing et al. hypothesized early mortality in sickle cell patients that could explain the young age of patients during a study in sub‐Saharan Africa, where they reported the death of more than half of children with sickle cell anemia before the age of 5 years [24]. These findings were also reported by Ousmane et al. [22]*.* The population was mostly underweight according to BMI (Table 1). The malnutrition to which the sickle cell patient is subject could be the explanation. Sombodi et al. also made this observation, as did Tuono et al. [25, 26]. History of infectious crisis, anemia, vaso‐occlusive, and history of cardiovascular disease have also been reported in patients during this study (Table 1). These described signs and symptoms are characteristic of sickle cell patients. In the review of Houwing et al. (2019), which provides a summary of the pathophysiology and management of SCD, the same observations were also described; this finding was also reported by Wonkam et al. [24, 27].

Among the *HP* genotypes, there is a higher frequency of the “*HP*1F” allele followed by “*HP*2.” Also, 14.09% of the population had the Hp 2‐2 phenotype. Several authors report identical distributions, i.e., with a predominance of the Hp 1‐1 phenotype, followed by the Hp 2‐1 phenotype, and, finally, the Hp 2‐2 phenotype. This is particularly the case for Olatunya et al. in Nigeria [28], Khalid and Khalil (2016) in Sudan [29], as well as Ostrowski et al. [8]. In contrast, our results are different from those of Pierrot‐Gallo et al. which report a predominance of the Hp 2‐1 phenotype followed by the Hp 2‐2 phenotype and finally the Hp 1‐1 phenotype [30]; results also differ from those of Kengne et al. (2021) who report a predominance of the Hp 2‐2 phenotype, followed by the Hp 1‐1 phenotype and finally the Hp 2‐1 phenotype [31]. This variability in outcome is also reported by Santos et al. in Brazil [9]. This variability in the result demonstrates the significant segregation of genes in the population; however, it still demonstrates a predominance of the Hp 1‐1 phenotype, followed by the Hp 2‐1 phenotype and, finally, the Hp 2‐2 phenotype as observed in the literature [9].

Oxidative stress assessment reports a general decrease in glutathione in the sickle cell population, although not statistically significant. Redox homeostasis is provided by a vital antioxidant, namely, glutathione [32]. The increase in oxidative stress in sickle cell patients increases the demand, leading to its irreversible loss, especially since it acts as a detoxin. Several authors attribute to it the important role in the removal of hydrogen peroxide and lipid‐like peroxides [33, 34]. In addition, our study reports a nonsignificant decrease in catalytic activity during sickle cell anemia. Authors also report a decrease in its activity during sickle cell anemia and oxidative processes [35]. Moreover, this study reports elevated levels of MDA during sickle cell anemia. MDA is one of the main parameters that provides information on the extent of cellular oxidative damage. Several authors report a significant increase in oxidative damage during sickle cell anemia [36]. This is the result of a high production of ROS released during the disease, causing significant oxidative damage. The ROS produced destroys and changes the structure of lipids, resulting in the formation of lipid peroxides, which are measured in MDA. In summary, despite the apparent influence of the oxidative parameters on inflammation, none was statistically significant.

Furthermore, during the study, patients with Hp phenotype 2‐2 had a considerable elevation of reduced glutathione compared to those of Hp 2‐1 phenotype and Hp phenotype 1‐1. In addition, catalase has a greater elevation in patients with Hp 1‐1, Hp 2‐1, and Hp 2‐2 phenotypes, respectively. The magnitude of oxidative stress would explain catalytic hypoactivity in patients, and conversely, the increase in catalase is to its H_2_O_2_ scavenging action [37]. Haptoglobin therefore appears to be one of the most important phenotypic modulators of sickle cell anemia, as it has the property of binding to plasma‐free hemoglobin, leading to the formation of the haptoglobin–hemoglobin complex for its elimination and thus preventing heme‐related oxidative damage [9].

In addition, glutathione concentrations were reported to be reduced in patients during this study. Indeed, glutathione is a cofactor of glutathione peroxidase for the reduction of H_2_O_2_ and is readily oxidized to glutathione disulfide (GSSG) during SCD by oxidizing compounds. [37]. Also, the reduction in concentrations of the latter according to studies would be closely related to the severity of the disease because they are the erythrocyte markers carried by sickle red blood cells in this case [38–40].

In addition, MDA in patients with Hp phenotypes 2‐2 shows a significant elevation compared to those of phenotype Hp 1‐1 and phenotype Hp 2‐1 (*p* = 0.008), suggesting that the Hp 2‐2 phenotype may promote an increase in MDA. The authors propose that alterations in the membrane structure of sickle cell erythrocytes promote increased production of ROS; ROS are important factors in membrane lipid peroxidation, which is more pronounced in patients with the Hp 2‐2 phenotype due to poor hemoglobin elimination [41]. In addition, excessive production of MDA exhibits additional toxic effects due to protein alteration, changes in the amino acid side chain, and membrane lipid structure, in which alterations could promote a loss of function of proteins, including antioxidant enzymes [39]. It is for this reason that MDA is considered a key marker of oxidative stress as it gives an idea of the extent of oxidative damage [6].

The TAC reported during this study is higher in sickle cell patient phenotype Hp 1‐1 than other total phenotypes (*p* = 0.062), which would suggest that the Hp 2‐2 phenotype of haptoglobin is in favor of a decrease in oxidative capacities and thus promotes oxidative stress. At the same time, several authors report a decrease in TAC in SS sickle cell patients [16]. Indeed, the TAC is a reflection of the reducing property of the individual nonprotein antioxidant and therefore has the advantage of giving general information on the antioxidant power. Its elevation is in response to an excessive presence of reactive oxygen species (ROS) in the blood [13]. Furthermore, its reduction is the result of higher oxidative stress in patients with the *HP* 2‐2 phenotype.

Finally, in our study, the OSI was higher in patients with the Hp 2‐2 phenotype than in those with the Hp 2‐1 and Hp 1‐1 phenotypes, suggesting that they are more affected by oxidative stress. This observation was also reported by several authors [16]. Furthermore, although oxidative stress is associated with inflammation, no significant difference was observed between patients with inflammation and those without during this study.

Although this study demonstrates that haptoglobin is a major contributor to the worsening pathophysiology of SCD, it has limitations, including a relatively small sample size, which limited the ability to draw more conclusive results regarding the influence of the haptoglobin 2 phenotype on oxidative stress dysregulation in patients with SCD.

## 5. Conclusion

The purpose of this study was to describe the relationships among iron, oxidative stress, and the genetic polymorphism of haptoglobin in sickle cell anemia patients with inflammation, and to explore how these factors are associated with the pathophysiology of the disease. It shows a higher frequency of phenotype Hp 1‐1 and less important phenotype Hp 2‐2 in homozygous Cameroonian sickle anemia patients. On the other hand, it notes the important role of iron in the occurrence of oxidative stress and demonstrates a greater association of the Hp 2‐2 phenotype of haptoglobin with the imbalance of the oxidative balance in favor of the increase of pro‐oxidants and a decrease in antioxidants, unlike the Hp 1‐1 phenotype. Observations made from this study suggest that haptoglobin is a major contributor to the worsening pathophysiology of sickle cell anemia; however, a study of a larger size would allow for more conclusive results.

## Disclosure

All authors have read and approved the final version of the manuscript.

## Conflicts of Interest

The authors declare no conflicts of interest.

## Author Contributions

Romaric Tuono De Manfouo made significant contributions to this study, including data collection, biological analysis and interpretation of biological data, statistical analyses, and wrote the first version of this manuscript. Jean Paul Chedjou and Wilfried Mbatcham supervised the molecular biology stage. Josué Simo Louokdom edited and corrected the first manuscript and made an important contribution to the statistical analysis of the data. Prosper Cabral Biapa Nya, Constant Anatole Pieme, and Claude Tayou Tagny designed and supervised the study and contributed to the interpretation of the data and the final writing of the manuscript. Romaric Tuono De Manfouo had full access to all of the data in this study and takes complete responsibility for the integrity of the data and the accuracy of the data analysis.

## Funding

This research did not receive any specific grant from funding agencies in the public, commercial, or not‐for‐profit sectors.

## Supporting Information

Supporting Information. It is made up of tables Table S1, S2 representing Primers and nucleotide sequences used and PCR primer set, respectively. Figures (S1), (S2), (S3), (S4), (S5), (S6), (S7), (S8), (S9) represent catalase activity and haptoglobin phenotypes, malondialdehyde and haptoglobin phenotypes, total antioxidant capacity and haptoglobin phenotypes, reduced glutathione and haptoglobin phenotypes, OSI index and haptoglobin phenotypes, catalase activity in the presence or absence of inflammation, oxidative stress index in the population in the presence or absence of inflammation, reduced glutathione in the population in the presence or absence of inflammation, and antioxidant capacity in the presence or absence of inflammation, respectively.

## Supporting information


**Supporting Information** Additional supporting information can be found online in the Supporting Information section.

## Data Availability

The data used to support the findings of this study are available from the corresponding author upon request. The authors confirm that the data supporting the findings of this study are available within the article and/or its supporting information.
