# Supplementary material for: Iron, Oxidative Stress, and Haptoglobin Gene Polymorphism in Sickle Cell Disease Patients With Inflammation in Cameroon: An Analytical Cross‐Sectional Study
Source: Biochem Res Int. 2025 Dec 28;2025:5303373. doi: 10.1155/bri/5303373 (PMC12745657; doi:10.1155/bri/5303373)
Supplement: Supplementary file 1 — Supporting Information Additional supporting information can be found online in the Supporting Information section. [file BRI-2025-5303373-s001.docx]

1. **Supplementary Material**

**Table S1: Primers and nucleotide sequences used**

| **Primers** | **Nucleotide Sequence (5’ 3’)** |
| --- | --- |
| F3 | CAGGAGTATACACCTTAAATG |
| S2 | TTATCCACTGCTTCTCATTG |
| C42 | TTACACTGGTAGCGAACCGA |
| C72 | AATTTAAAATTGGCATTTCGCC |
| C51 | GCAATGATGTCACGGATATC |

**Table S2: PCR primer set**

| Reactions | Jeux d’amorces | Allèles cibles | Taille prévue (bp) |
| --- | --- | --- | --- |
| Reaction 2 | F3-C42 | *Hp^2^* | 935 |
| Reaction S | C51-S2 | *Hp^1S^* | 1,2k |
| Reaction F | F3-C72 | *Hp^1F^* | 1,4k |

1. ***Supplementary Results***

The following figure (S1) describes the catalase activity in the population according to the haptoglobin phenotypes:


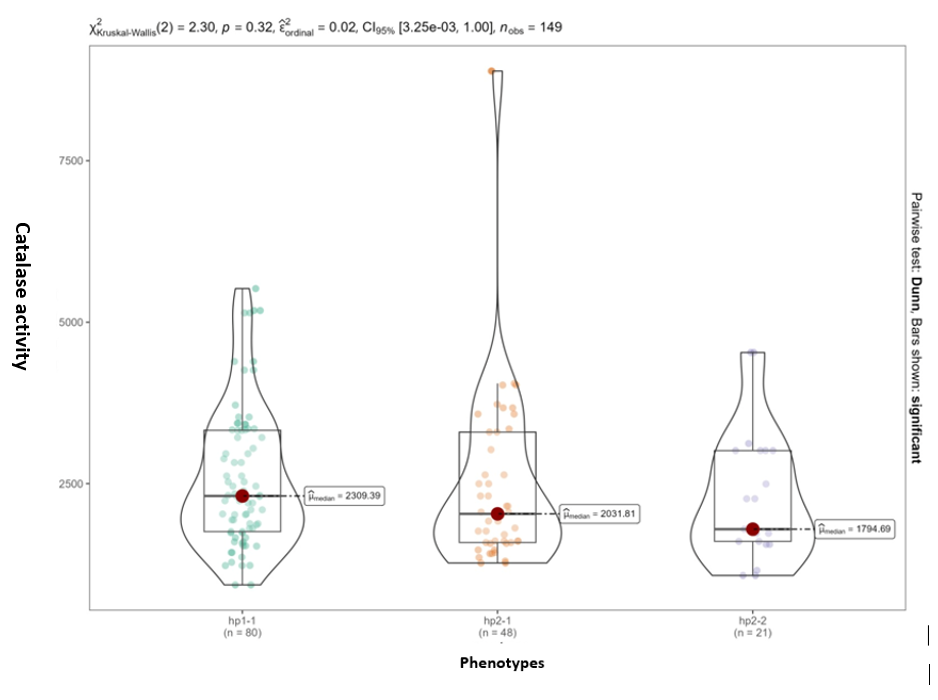


***Legend****: Hp1-1 : Haptoglobin phenotype 1-1 ; Hp 2-1 : Haptoglobin phenotype 2-1 ; Hp2-2 : Haptoglobin phenotype 2-2 ;*

**Figure S1: Catalase activity and haptoglobin phenotypes**

The following figure (S2) describes MDA in the population according to the haptoglobin phenotypes:


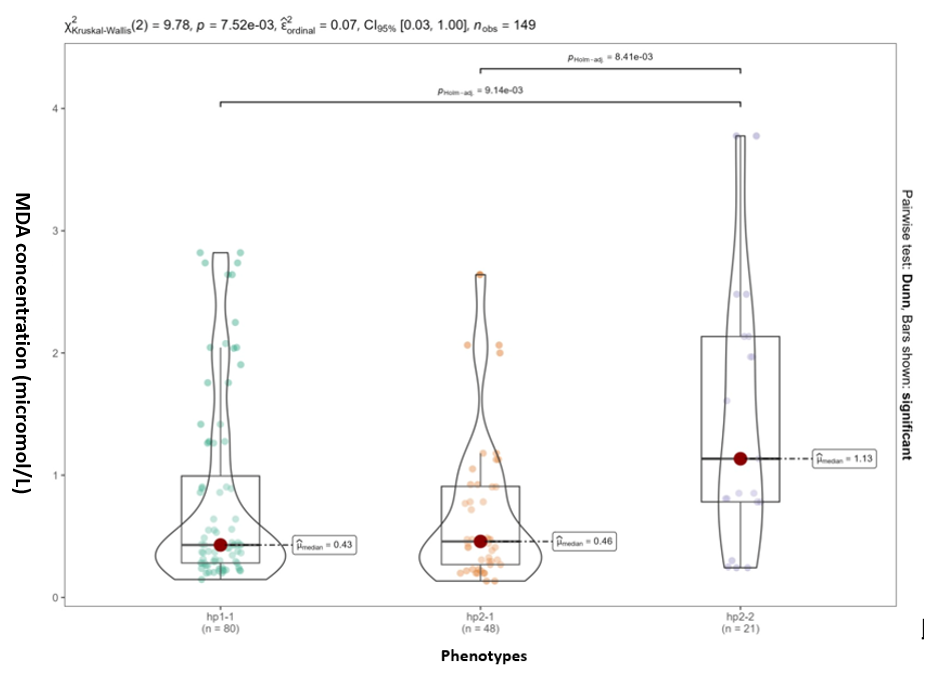


***Legend****: Hp1-1 : Haptoglobin phenotype 1-1 ; Hp 2-1 : Haptoglobin phenotype 2-1 ; Hp2-2 : Haptoglobin phenotype 2-2 ; MDA : Malondialdehyde*

**Figure S2: Malondialdehyde and haptoglobin phenotypes**

The following figure (S3) describes the Total Antioxidant Capacity in the population according to the haptoglobin phenotypes:


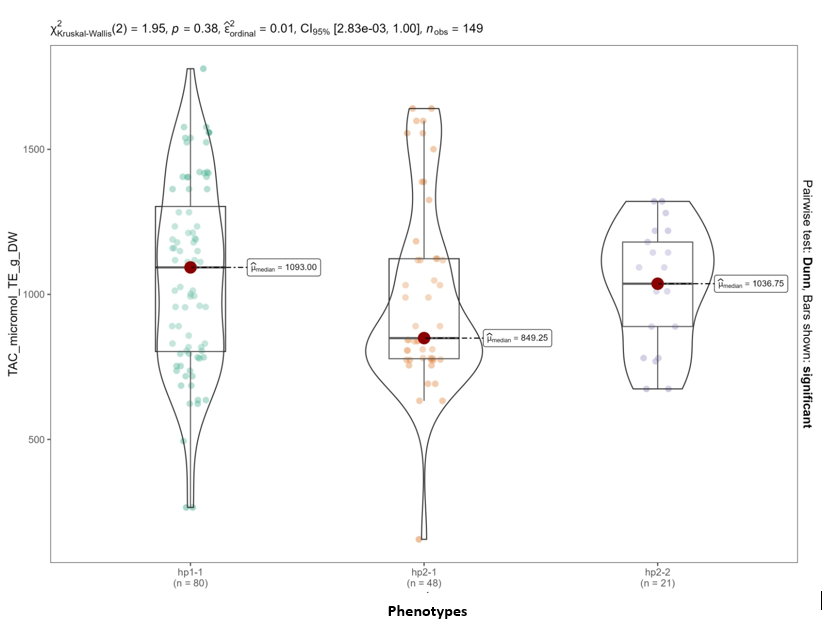


***Legend****: Hp1-1 : Haptoglobin phenotype 1-1 ; Hp 2-1 : Haptoglobin phenotype 2-1 ; Hp2-2 : Haptoglobin phenotype 2-2 ; TAC : Total Antioxidant Capacity*

**Figure S3: Total Antioxidant Capacity and Haptoglobin Phenotypes**

The following figure (S4) describes reduced glutathione in the population according to the haptoglobin phenotypes:


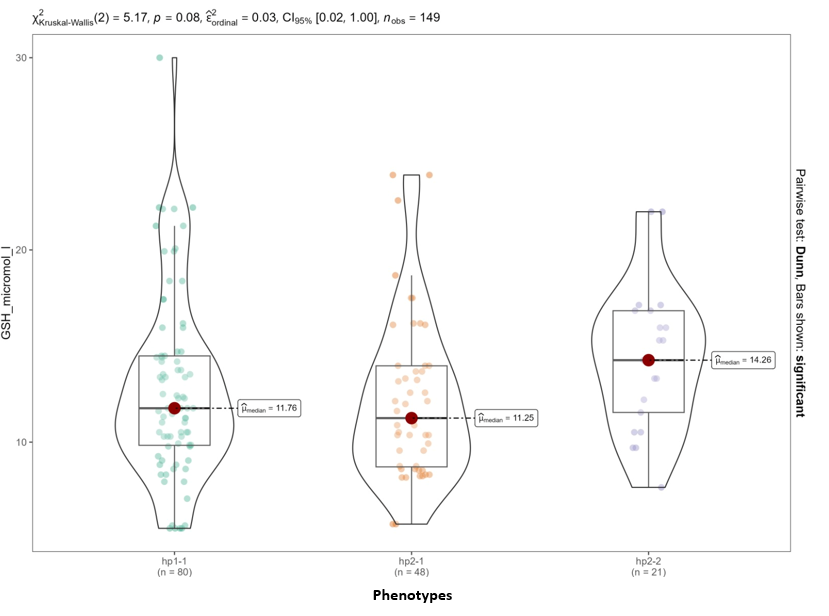


***Legend****: Hp1-1 : Haptoglobin phenotype 1-1 ; Hp 2-1 : Haptoglobin phenotype 2-1 ; Hp2-2 : Haptoglobin phenotype 2-2 ; GsH : Reduced glutathione*

**Figure S4**: **Reduced glutathione and haptoglobin phenotypes**

The following figure (S5) describes the OSI in the population according to the haptoglobin phenotypes:


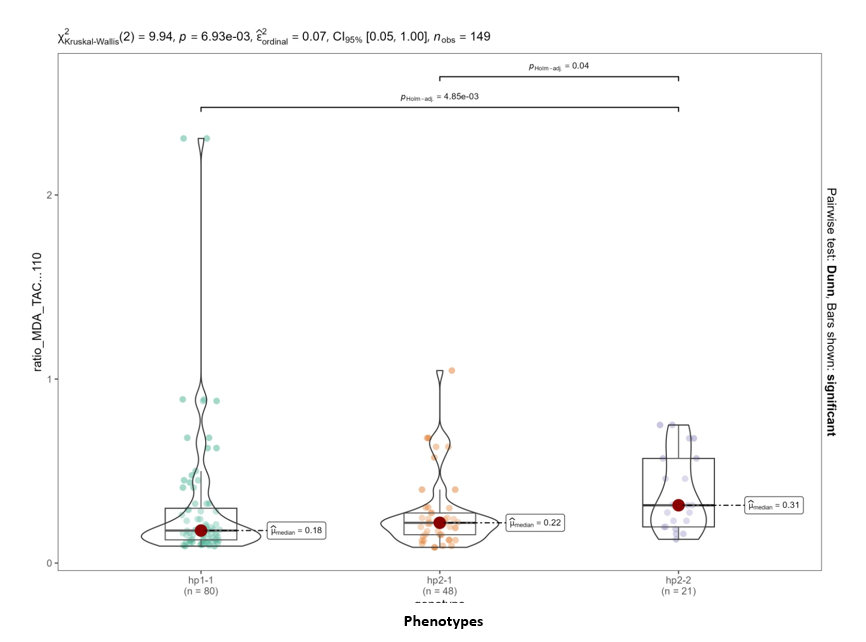


***Legend****: Hp1-1 : Haptoglobin phenotype 1-1 ; Hp 2-1 : Haptoglobin phenotype 2-1 ; Hp2-2 : Haptoglobin phenotype 2-2 ; ratio MDA/TAC : ratio Malondialdehyde/Total Antioxidant Capacity*

**Figure S5: OSI Index and Haptoglobin phenotypes**

The following figure (S6) describes the catalase activity in the population in the
presence or absence of inflammation:


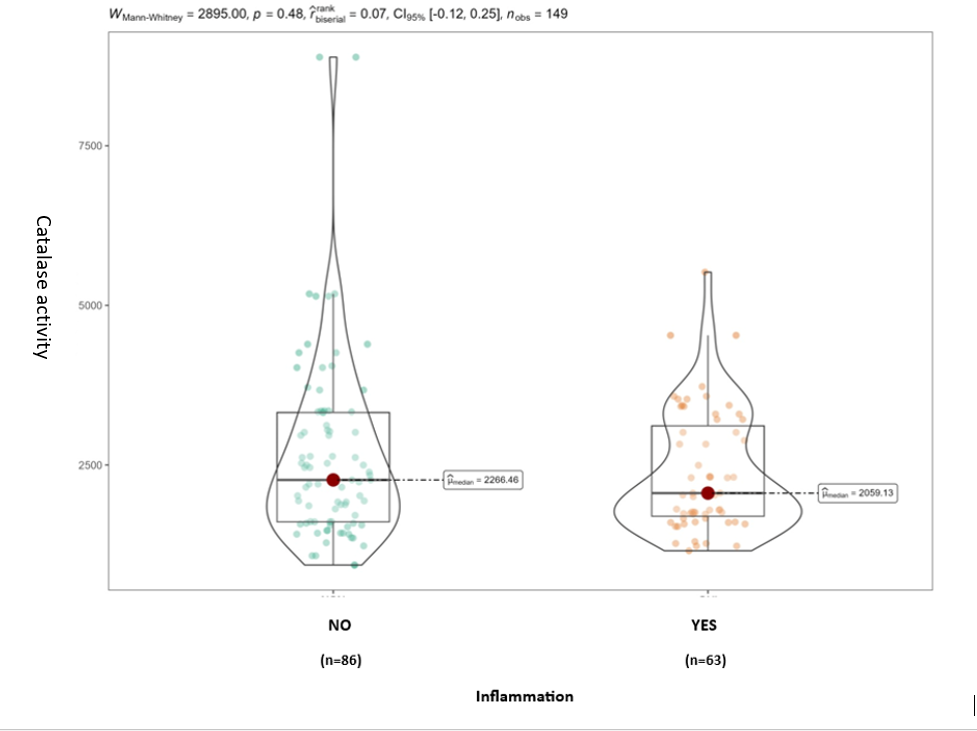


**Figure S6: catalase activity in the presence or absence of inflammation:**

The following figure (S7) describes the Oxidative Stress Index in the population in the
presence or absence of inflammation:


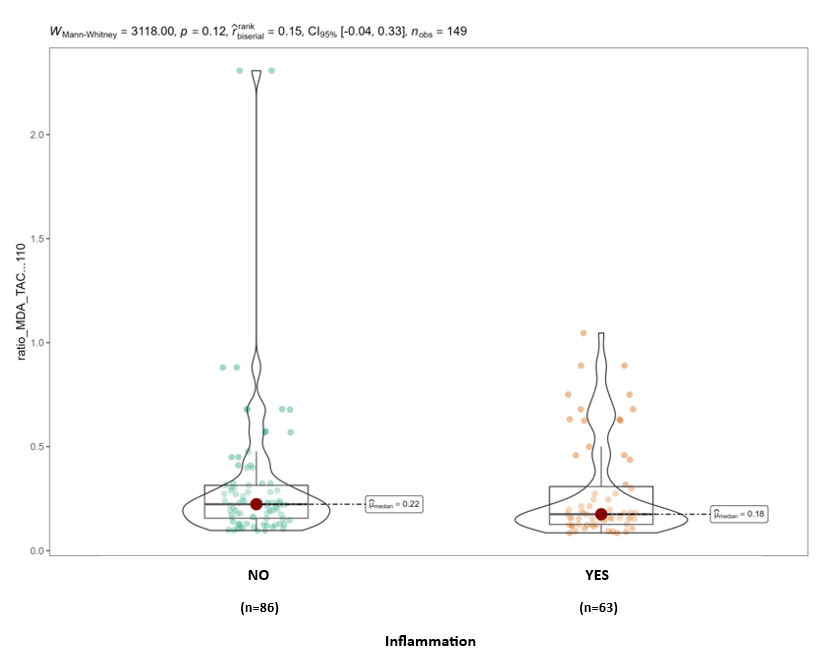


***Legend****: ratio MDA/TAC : ratio Malondialdehyde/Total Antioxidant Capacity*

**Figure S7: Oxidative Stress Index** **in the population in the
presence or absence of inflammation**

The following figure (S8) describes reduced glutathione in the population in the
presence or absence of inflammation:


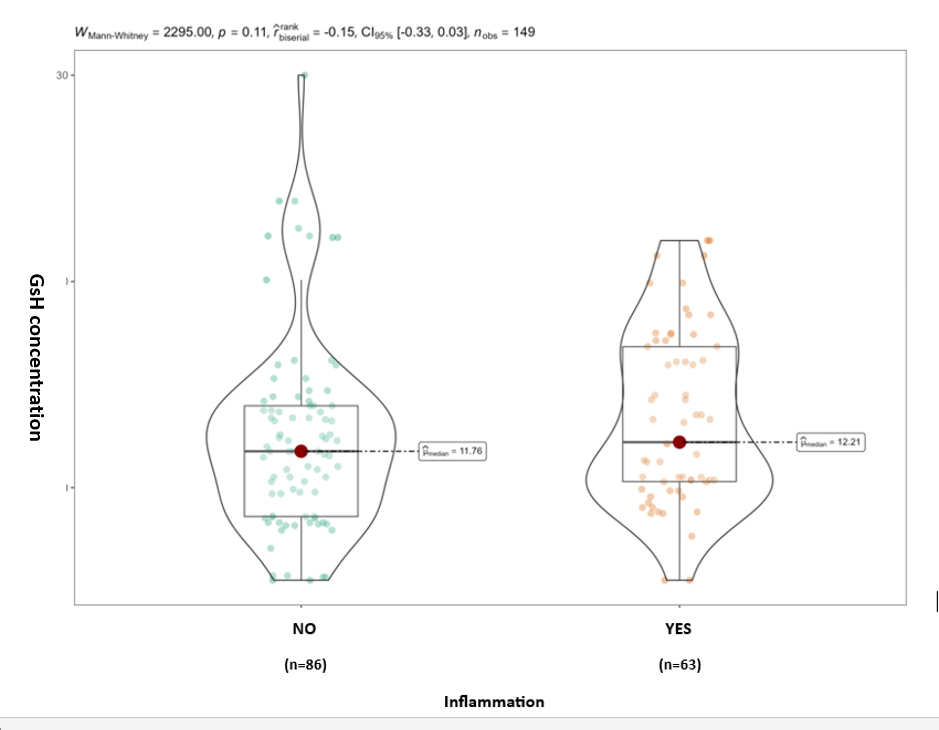


***Legend****: GsH : Reduced glutathione*

**Figure S8: Reduced glutathione in the population in the
presence or absence of inflammation**

The following figure (S9) describes the Total Antioxidant Capacity in the
presence or absence of inflammation:


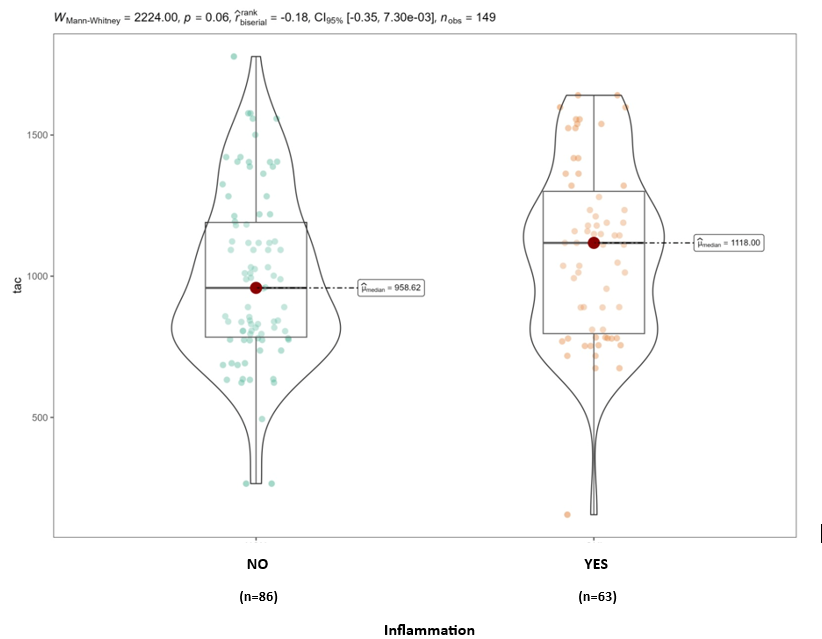


***Legend****: TAC : Total Antioxidant Capacity*

**Figure S9: Total Antioxidant Capacity in the
presence or absence of inflammation:**
